# Eco-Friendly CuO/Fe_3_O_4_ Nanocomposite synthesis, characterization, and cytotoxicity study

**DOI:** 10.1016/j.heliyon.2024.e27787

**Published:** 2024-03-08

**Authors:** Poonam Dwivedi, Abdul Malik, Hafiza Zumra Fatima Hussain, Indu Jatrana, Khalid Imtiyaz, M.M. Alam Rizvi, Md Mushtaque, Azhar U. Khan, Mahboob Alam, Mohd Rafatullah

**Affiliations:** a Department of Chemistry, School of Basic Sciences, Jaipur National University, Jaipur, 302017 (Rajasthan) India; b Department of Pharmaceutics, College of Pharmacy, King Saud University, Riyadh 11451, Saudi Arabia; c Department of Environmental, Biological and Pharmaceutical Science and Technology (DISTABiF), University of Campania ‘Luigi Vanvitelli’ Via Vivaldi 43, 81100 Caserta, Italy; d Department of Biosciences, Jamia Millia Islamia, New Delhi, 110025, India; e Department of Chemistry, Millat College (A constituent colle ge of Lalit Narayan Mithila University), Darbhanga, Bihar, India; f Division of Chemistry and Biotechnology, Dongguk University, 123, Dongdaero, Gyeongju-si 780714, Republic of Korea; g Environmental Technology Division, School of Industrial Technology, Universiti Sains Malaysia, 11800 Penang, Malaysia

**Keywords:** Nanocomposite, pumpkin seeds extract, MTT-Assay

## Abstract

The current study report a convenient, simple, and low cost approach for the biogenic synthesis of CuO/Fe_3_O_4_ nanocomposites (NCs) from pumpkin seeds extract and their vitro cytotoxicity. The characterization of finally obtained CuO/Fe_3_O_4_ nanocomposites (NCs) performed using UV–Visible, FT-IR, XRD, XPS, GC-MS, SEM-EDX and TEM analysis. The formation and elemental analysis were determined using the energy-dispersive X-ray (EDX) microanalysis technique. The formation of rod-like monoclinic and spherical, having size range 5 nm–20 nm confirmed by scanning electron microscope (SEM) and transmission electron microscopy (TEM) respectively. Finally, the MTT assay of the synthesized composites was evaluated for toxicity against cancerous cell lines HCT-116 (Colon cancer cell) and A549 (human lung adenocarcinoma cell). The synthesized composite material showed moderate (IC_50_ = 199 μg/mL) to low (IC_50_ = 445 μg/mL) activity against HCT-116 and A549 cell lines, respectively.

## 1 Introduction

Nanocomposites with desirable features from more than two phases have recently produced significant result and developed the scientific interest having their novel property and distinctive design [1]. As a result, they have been exploited in various fields, including catalysis, sensing, bioengineering, and renewable energy, because they can circumvent single-phase or micro-composites' limitations [[2], [3], [4], [5]]. In the past, composite nanomaterials have been made by combining secondary phases with existing components' internal or external surfaces. These techniques include chemical vapor deposition, hydrothermal, sol-gel, solution mixing, and sol-gel [6]. However, most of these processing methods still have issues managing chemical composition, architecture, and stoichiometry.

Traditionally, Plants have been exploited on a large scale in medicine, biomedical, textile, cosmetic and food. In the previous year's plants use as medicine in Ayurveda, to cure the diseases, the biomedical applications of plants enhanced rapidly due to advancement technological and temporal platform [[7], [8], [9]]. The phytochemicals found in plants, which are among the most exciting elements of plants since they include properties like antibacterial, antitumor, anti-ageing, and others [[10], [11], [12]] are primarily responsible for these biological uses. Researchers came to know more applications of phytochemicals which compelled them to explore. Recently, an innovative multidisciplinary study invented a method for developing ano-materials from plant extracts' phytochemicals that is more environmentally benign than traditional procedures and does not require. Pumpkin is a medicinally important plant with many bioactive constituents such as, alkaloids, flavonoids, palmitic, oleic and linoleic acids [13]. In the case pumpkin seeds exhibited useful nutrients and also nutraceuticals like cucurbitacins, tocopherols, phenolic compounds, unsaturated fatty acids, phytosterols and amino acids and play a important role in our healthy life and well-being [14]. The reason for selecting the pumpkin seeds extract was the presence of high active bio-constituents and our experience of successfully biosynthesizing various nanoparticles showing potent anticancer activities [[15], [16], [17], [18]]. Micro-composite and nanocomposite materials are acquired in different ways such as polymer matrix nanocomposites, metal matrix nanocomposite and ceramic matrix nanocomposite [1].

Nanocomposite materials are known to exhibit a wide spectrum of biological applications such as anti-microbial [19,20], anti-cancer [20,21]. In order to explore nanocomposite materials, attempts are being made by researchers. In this pursuits, Le et al. (2021), investigated the fabrication of Fe_3_O_4_/CuO@C composite and their photo-catalyst for degradation [22]. Todorova et al. (2010) reported the formation of mesoporous CuO–Fe_2_O_3_ composite and use as catalysts for oxidation of n-hexane [23]. Ghassemi et al. (2018) reported CuFe_2_O_4_/CuO by cathodic electro approach applies for high performance super-capacitor purpose [24]. Panthawan et al. (2022), reported Cu–Fe oxide composite films use of a one-step sparking approach and also observed their photo-catalytic effectiveness under the visible light [25]. The previous year's survey revealed that generally novel nonmaterial exhibited large surface area and has wide range of application in different fields, Ngoc and Vu (2022) have fabricated CuO.Fe_3_O_4_/silica composite using rice husk and their role to increase the Fenton-like catalytic degradation of tartrazine in proper pH range [26]. Xu et al. (2022) reported CuO–Fe_2_O_3_.MXene composite and use for atrazine degradation mechanism, performance and coexisting matter influence [27]. As per literature survey, numerous research articles have been reported in biosynthesis of different CuO/Fe_2_O_3_, Cu/reduced graphene oxide/Fe_2_O_3_, and magnetic chitosan-copper nanocomposite from different species [[28], [29], [30], [31], [32], [33]], also seen their action on thermal decomposition of ammonium perchlorate [34], carbon nanotubes (CNTs) [[35], [36], [37], [38], [39]]. In the continuation, a green approach for synthesis of CuFeO_2_ and CuO composite and their effect as photo-catalyst films and utilizing for the production of liquid format from CO_2_ and water was also reported [40]. Kiziltas and Tekin (2020) reported the fabrication and characterization of Fe_3_O_4_@CuO composite and use as photo-catalysts and applied to ascertainment of photo-catalytic activity on Rhodamine B [41]. In addition to this, Guo and Fan (2021) reported the preparation of Fe_3_O_4_–CuO bimetallic composite/functionalized CNTs and using as modified carbon paste electrode treated to identification of dexamethasone as a doping agent in sport [42]. In view of wide applications and importance, we herein report the a novel biogenic synthesis of CuO/Fe_3_O_4_ NCs using pumpkin seeds extract act stabilizing and reducing agent and its cytotoxicity against A549 (human lung adenocarcinoma cell) and HCT-116 (Colon cancer cell) respectively.

## 2 Materials and methods

All the required chemical using in the investigation obtained from Merck India Ltd and utilized in without purification.

### 2.1 Preparation of extract

In a 250 mL round bottom flask, 20 g of dried biomaterial was soaked in 50 mL deionized water. The mixture was refluxed for an hour at 70 °C. Afterwards, the suspension was left to room temperature for 30 min then filter through Whatmann's filter paper and kept in store or refrigerator for further studies.

### 2.2 Biosynthesis of CuO/Fe_3_O_4_ NCs

The CuO/Fe_3_O_4_ NCs was prepared through a green route. During the process, 1.1 g copper acetate dehydrates was add in a round bottom flask containing 50 mL DI water and stirred the reaction mixture. Subsequent to this, 1.62 gm ferric chloride was dissolved into the flask. The suspension was heated on hot plate at 80 °C then 40 mL of plant extract was added slowly into it. After 30 min of heating and vigorous stirring, solution medium was made alkaline (pH = 10) by adding 2 mL of 1 N sodium hydroxide solution. After 2 h of continuous stirring, the solution color changes from reddish brown to blackish brown, confirming the formation of CuO/Fe_3_O_4_ NCs. After cooling, the suspension centrifuged 10,000 rpm for 10 min and wash four times ethanol to remove unwanted extract and NaOH. The washed sample was dried in hot air oven at 70 °C for 6 h and then was calcined at 350 °C. Finally, the obtained Nanocomposite of CuO/Fe_3_O_4_ was stored in sample tubes for characterization and other studies.

### 2.3 Nanocomposite characterization

The bio reduction of CuO/Fe_3_O_4_ NCs were monitored using UV–vis spectrum (PerkinElmer Lambda750) at MNIT, Jaipur, The absorption maxima was scanned by its at 1 nm ranging from 200 to 800 nm. The size and morphology of CuO/Fe_3_O_4_ were measured by TEM (TECNAI G-20) and SEM (Nova Nano FE-SEM 450 FEI). The brown colour powder material sample of biogenic synthesized CuO/Fe_3_O_4_ NCs was analyzed through Fourier transform-infrared (FT-IR) spectra in the range of 4000-400 cm^−1^ with with KBr pellet technique. Similarly, EDX spectroscopic explain the elemental composition of CuO/Fe_3_O_4_ NCs and the particles dried on a carbon-coated grid and applied on SEM instrument with thermo EDX attachment. The crystal phase of the synthesized materials was detected on an X-ray diffractometer (Bruker D8, IIT, Roorkee) operated at 40 kV and 30 mA with Cu/Kα radiation with 2*θ* ranging from 10° to 80°. The bioconstituents of alcoholic extract of pumpkin seeds were examined by using gas chromatography coupled to a mass spectrometer, (GC–MS). A 30 mm × 0.25 mm id HP-column with a 0.25 μm film thickness was filled with the alcoholic extract (Agilent technologies 6890 N JEOL GC Mate II GC–MS model). An electron ionization system operating at an ionization voltage of 70 eV was employed for the purpose of detection. As a carrier gas, pure helium gas (99.999%) was employed at a steady flow rate of 1 mL/min. The ion-source temperature was intended to be 200 °C, while the injector temperature was kept at 250 °C. The oven was set to start at 110 °C and climb by 10 °C each minute to 200 °C. The NIST database was used to determine the name, molecular weight, and structure of phytoconstituents and fatty acids [43].

### 2.4 Cytotoxicity studies

#### 2.4.1 Cell culture

Both the human lung adenocarcinoma cell A549 and the colon cancer cell HCT-116 were purchased from the National Curator of Cell Sciences (NCCS) in Pune, India. According to Gupta et al. (2006), cells were grown in Dulbecco's modified Eagle's medium (DMEM) with 10% fetal bovine serum, 100 units/mL of penicillin, 100 g/mL of streptomycin, and 2.5 g/mL of amphotericin B at 37 °C and 80% relative humidity with 5% CO_2_ [44]^.^

#### *2.4.2* *MTT*-assay

116 and A549 cancer cell lines were used to examine the CuO/Fe_3_O_4_ NCs nanocomposite material using the MTT-assay method and the MTT-(3-(4,5-dimethylthiazole-2-yl)-2,5-diphenyl tetrazolium bromide, M2128 Sigma Aldrich) dye. Using the MTT-assay, any chemical substances in vitro cytotoxicity can be evaluated [45]. 1 × 10^4^ cells/well on a 96-well plate (150 L/well) of cells were planted. Following an overnight incubation, cells were exposed to various quantities of nanocomposite material for 24 h. A 24-h treatment session was followed by the removal of the medium. Then, it was exposed to 20 L of MTT solution (5 mg/ML in phosphate saline buffer) for 4 h. To create formazan crystals, mitochondrial enzymes were reduced. After that, they were dissolved in DMSO (150 L/well), and an absorbance reader (i-Mark, BIORAD, S/N 10321) was used to quantify the absorbance at 570 nm. The % viability was calculated using the relative absorbance of treated and untreated control.

### 2.5 Statistical analysis

Experiments were performed in triplicate and results are expressed as mean ± standard error of the mean (SEM). IC_50_ values were determined by fitting the model with linear regression using Origin and Excel software. Data were analyzed using one-way analysis of variance (ANOVA). Statistical significance was considered when p < 0.05.

## 3 Results & discussion

### 3.1 UV–visible spectroscopy

**Fig. 1:**
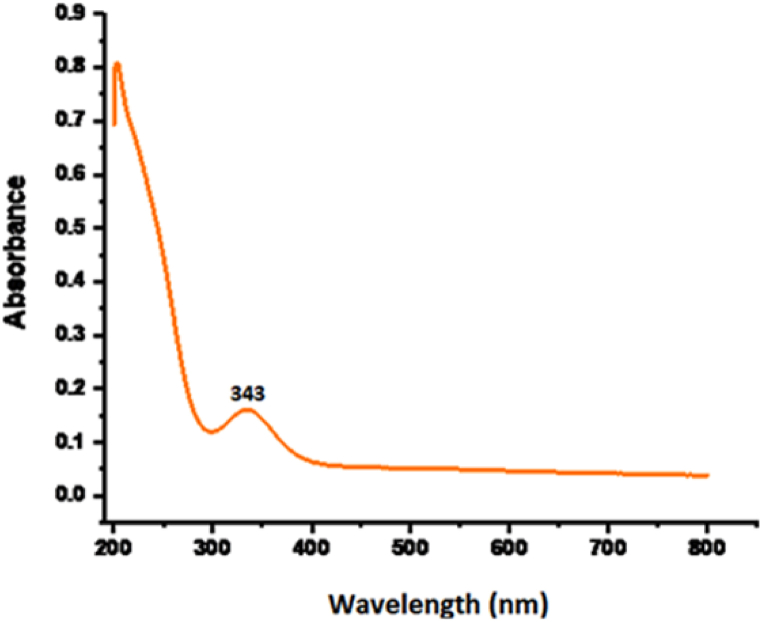
UV–visible spectrum of CuO/Fe_3_O_4_ NCs synthesized from seeds extract of pumpkin.

The biogenic synthesized ^CuO/Fe^_3_^O^_4_ nanocomposite observed a significant absorption maxima appeared at 343 nm in UV–vis spectroscopy as shown in Fig. 1, ^which may be due to CuO's surface Plasmon resonance (SPR) effect in the synthesized CuO/Fe^_3_^O^_4_^NC^ inferred the good optical properties ^of biogenic CuO/Fe^_3_^O^_4_^NC, confirmed the formation by similar studies^ [46]^.^

### 3.2 FT-IR spectroscopy

**Fig. 2:**
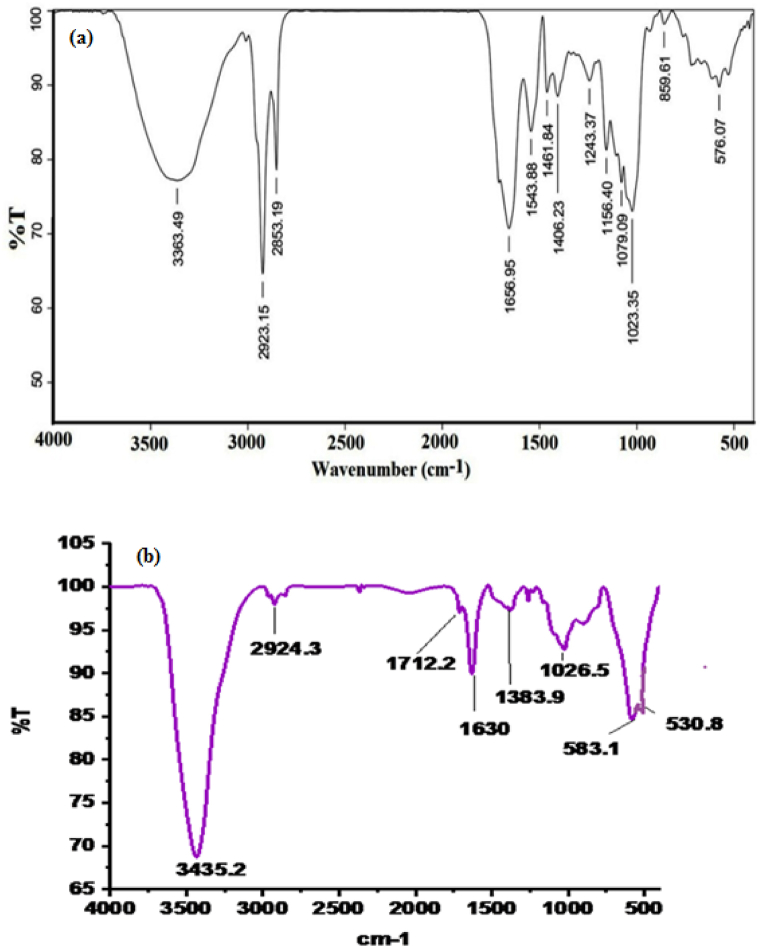
FT-IR Spectrum (a) extract of pumpkin seeds and (b) CuO/Fe_3_O_4_ NCs.

The FTIR data favor the formation of CuO/Fe_3_O_4_ NCs obtained by green route as shown in Fig. 2(a) is extract of pumpkin seeds, describe the peak at 3335 cm^−1^ assign O–H stretching vibration of the hydroxyl group, 2922 and 2853 cm^−1^ absorption assign C–H stretching of alkane and aromatic ring, the prominent peal observed at 1656 cm^−1^ C=O stretch α, β-unsaturated ketone groups or aromatic rings double bond, 1543 cm^−1^ (amide II). The 1023, 1461, 1406 cm^−1^ can be assigned to C–O and C–C stretching of alcohols respectively [18]. The band appear at 1156 cm^−1^ describe the presence of C–N stretching of aliphatic amine, the peak observed at 576 cm^−1^ was assigned for C–Cl stretching. Hence the functional biomolecules are fatty acids, phenolic compound and amine groups involved in the reduction process. Fig. 2(b) the FTIR of CuO/Fe_3_O_4_ NCs the possible band at 3435.2 cm^−1^ observed in bioactive constituents as phenolic compound and responsible foe peaks near capping and efficient stabilization of the CuO/Fe_3_O_4_ NCs. An absorption bands observed in the ^range^ 2900-2800 cm^−1 describe^ the C–H stretching vibrations of bioactive constituents interacted with nanocomposite. Hence in the continuation, another absorption band seen at 1712.2 cm^−1^ appear due to aryl ketone and 1630 cm^−1 may^ be H–*O*– H bending vibrations [47]. The IR peak at 1383.9 cm^−1^ in the spectrum is ascribed to the –C–O–C− moiety [48] and this can be attributed to the fatty acids present in the extract, as substantiated by a complementary GCMS study. The band appeared at 1026.5 cm^−1^ assign to C–O in functional groups. The absorption band appear at 530.87 cm^−1^ and 583.10 cm^−1^ stretching vibrations support of Cu–O and Fe–O bond and favor the formation of CuO/Fe_3_O_4_ NCs respectively [49,50].

### 3.3 Powder XRD analysis

**Fig. 3:**
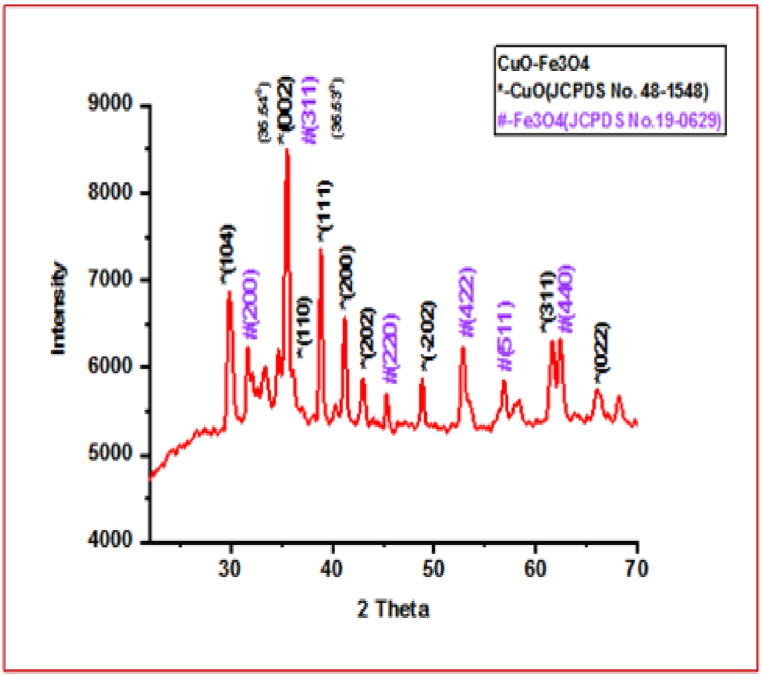
XRD pattern of CuO/Fe_3_O_4_ NCs synthesized from seeds extract of pumpkin.

The crystallinity of biosynthesized CuO/Fe_3_O_4_ NCs was examined by powder X-ray diffraction analysis (XRD) as shown in Fig. 3. The green produced CuO/Fe_3_O_4_ NCs have characteristic XRD peaks at 2*θ* = 29.93°, 35.54°, 36.14°, 38.79°, 41.12°, 42.97°, 48. 89°, 61.60° and 66.13°, which corresponds to (104), (002), (110), (111), (200), (202), (−202), (311),and (022) crystalline planes of monoclinic CuO, respectively (JCPDS Card No. 48–1548) [51,52]. The significant peaks of Fe_3_O_4_ in XRD pattern of sample are observed at 2*θ* = 31.61°, 35.53°, 45.33°, 53.41°, 56.89°, and 62.39°, assigned to the respective (200), (311), (220), (422), (511), and (440) crystallographic planes of cubic phase magnetite (JCPDS No.19-0629). These PXRD observations confirmed that the green synthesized Nanocomposite was CuO/Fe_3_O_4_. In addition, the crystallite size nature generated from Nano-crystalline sample was calculated from X-ray line broadening using the Debye–Scherrer formula, D = 0.9 *λ*/*β*cos *θ.* [53,54], where *β*—the line broadening at half maximum intensity (FWHM in radians on the 2*θ* scale) *λ*—the X-ray wavelength (1.5406°A), *θ*—the Bragg angle, and *D*—crystallite size in nm. The average particle size of CuO/Fe_3_O_4_ NCs was calculated using the Scherer equation, which was found to be 19.01 nm in the crystalline plane (311).

### 3.4 Morphological study

**Fig. 4:**
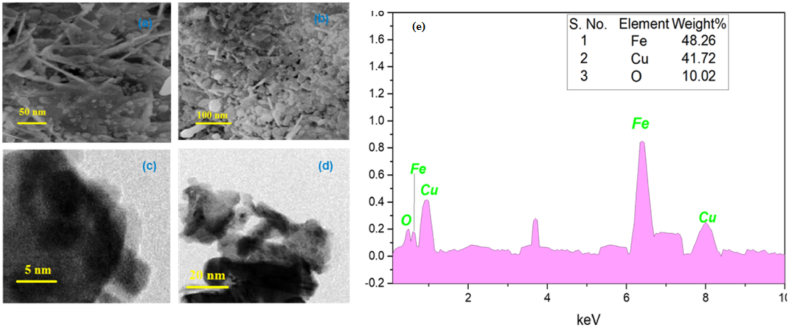
**(a) and (b)** SEM image of CuO/Fe_3_O_4_ NCs at different magnifications of micrometer, (c) and (d) TEM image of CuO/Fe_3_O_4_ NCs and (e) EDX spectrum and chemical composition of CuO/Fe_3_O_4_.

The SEM image of biosynthesized material obtained from pumpkin seed extract and found to be gathered on the surface as shown in Fig. 4 (a & b), two different shape particles, rod-like monoclinic CuO and spherical-shaped cubic Fe_3_O_4_ are clearly seen. SEM also revealed that particles are self-associated with various nano-spheres, resulting in a rough and porous surface. TEM images Fig. 4 (c & d) analyze expose that the biogenic synthesized CuO/Fe_3_O_4_ NCs exhibited the size range to 5 nm and 20 nm having in spherical in shape respectively.

Moreover, The EDX data analyzed and evaluate the provide composition of the materials obtained from pumpkin seeds extract. Furthermore, from energy dispersive X-ray spectrum (Fig. 4 e), Cu, Fe and O emission peaks are clearly recognized with weight percentage 48.26%, 41.72% and 10.02%, respectively.

### 3.5 GC-MS analysis

**Fig. 5:**
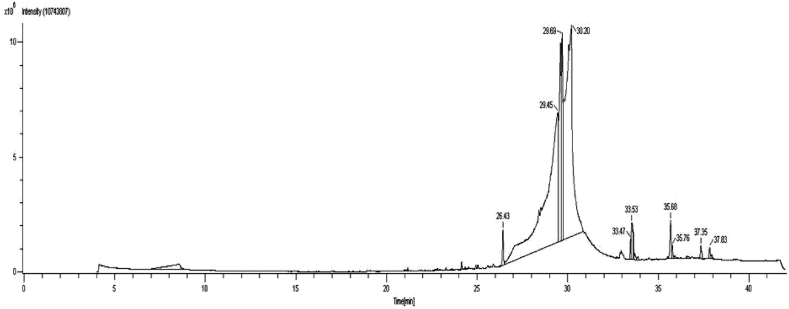
GC–MS chromatogram of pumpkin seeds extract.

Eight peaks were detected in the pumpkin seeds extract by GC–MS analysis as shown in Fig. 5. NIST database was used for interpretation. Hexadecanoic acid, ethyl ester, oleic acid, 9-Octadecenoic acid, Cis-Vaccenic acid, 9, 12-Octadecadeinoic acid, 7-Heptadecene, 1-chloro, and 6,11-Dimethyl-2,6,10-dodecatrien-1-ol were among the chemicals found in the extract of pumpkin seeds, as indicated in Table 1. It was determined that the eluted chemicals were methyl esters of fatty acids, flavone, and unsaturated fatty acid (oleic acid). The physiologically active compounds that were obtained have medicinal potential for human health. 9, 12-Octadecadienoic acid was the main bioactive ingredient in the extract from C. maxima seeds. This substance has multiple biological properties, including being hepatoprotective, anti-inflammatory, antihistaminic, and anti-arthritic. It is crucial to the prostaglandin manufacture of cell membranes [55,56]. The presence of this chemical in the extract raised questions about the plant's possible therapeutic use. There was only a small amount of 9-Octadecenoic acid found in the C. maxima seed crude extract. According to Hagr et al. (2018), this chemical has strong promise as an antioxidant, anti-inflammatory, and anti-cancer agent [57]. The rate-limiting step in the absorption of cholesterol is cholesterol acyl transferase, which is inhibited by unsaturated fatty acids found in plant products [58]. Oleic acid is one of the fatty acids found in pumpkin seeds. The primary fatty acid in terms of percentage is oleic acid [59]. Oleic acid was detected in the pumpkin seed extract by GC–MS analysis. By means of the adaptive thermogenic effect, polyunsaturated fatty acids found in nuts and oil seeds inhibit the accumulation of fat in adipose tissues [60]. In addition to fatty acids, pumpkin seeds contain other non-nutritive substances that also have antihyperlipidemic qualities.

**Table-1:** GC–MS analysis of pumpkin seeds extract.

Retention time (mins)	Percentage Area (%)	Compound name	Molecular formula	Molecular weight
26.43	0.83	Hexadecanoic acid, ethyl ester	C_18_H_36_O_2_	284
29.45	38.54	Oleic acid	C_18_H_34_O_2_	282
29.69	6.93	9-Octadecenoic acid	C_18_H_34_O_2_	282
30.20	37.46	cis-Vaccenic acid	C_18_H_34_O_2_	282
33.47	0.416	9, 12-Octadecadeinoic acid	C_18_H_32_O_2_	280
33.53	1.19	9, 12-Octadecadeinoic acid	C_18_H_32_O_2_	280
35.68	1.14	Oleic acid	C_18_H_34_O_2_	282
35.76	0.29	7-Heptadecene, 1-chloro	C_17_H_33_Cl	272
37.35	0.30	6,11-Dimethyl-2,6,10-dodecatrien-1-ol	C_14_H_24_O	208
37.83	0.38	9, 12-Octadecadeinoic acid	C_18_H_32_O_2_	280

Analysis of the seed extract revealed a fascinating array of organic compounds, with Oleic acid at 29.45 min (retention time) being the most abundant component, as shown in Table 1. This is closely followed by cis-Vaccenic acid at 30.20 min (retention time), another important fatty acid. Three interesting peaks on the chromatogram at 33.47 min, 33.53 min and 37.83 min indicate the presence of different isomers of 9,12-octadecadienoic acid. This suggests that the extract may contain a variety of structurally similar molecules, each with its own unique properties and potential applications. In addition to these prominent peaks, the chromatogram also provides a glimpse of richer organic compounds. Fatty acids, esters, and other interesting molecules move across the baseline, hinting at the complex biochemical machinery at work in the seeds.

### 3.6 XPS analysis

**Fig. 6:**
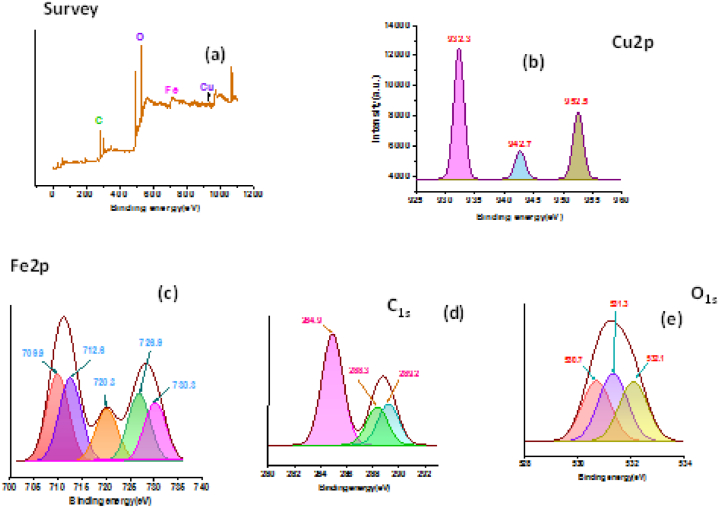
XPS analysis of CuO–Fe_3_O_4_ NC synthesized from seeds extract of pumpkin.

To show the chemical constitution of the surface of biosynthesized CuO/Fe_3_O_4_ NCs, XPS analysis was performed. The production of CuO/Fe_3_O_4_ NCs is confirmed by the presence of significant peaks at binding energies of 934 eV (Cu2p), 723 eV (Fe2p), 529 eV (O1s), and 283 eV (C1s), as shown in Fig. 6(a) which shows the whole scan spectra of CuO/Fe_3_O_4_. Fig. 6(b–e) displays the high resolution XPS spectra of Cu2p, Fe2p, O1s, and C1s.The spin-orbit doublet Cu2p3/2 and Cu2p1/2 peaks in the Cu2p spectra (Fig. 6(b)) were centered at binding energies of 952.5 and 932.3 eV, respectively. Cu 2p 1/2 level is represented by the shake-up satellite peak at 942.7 eV, which indicates the existence of Cu^2+^ ions in the sample. The binding energies of Fe2p3/2 and Fe2p1/2 of Fe_3_O_4_ were ascribed to the doublet for Fe2p in Fig. 6(c) at 710.9 and 727.1 eV, respectively [[61], [62], [63]]. Three peaks can be seen in the XPS spectra of C1s (Fig. 6(d)) at 284.9 eV (C–OH) [64], 288.3 eV (C=C) [62,65], and 289.2 eV (C=O) [66]. These peaks are thought to be caused by the active carboxylic group and alcoholic group present in fatty acids present in the leaf extract stabilizing CuO/Fe_3_O_4_ NCs [67]. Ferro-ferric oxide was identified as the peak position at 531.3 eV in the O1s spectra displayed in Fig. 6(e) for oxygen in Fe–O of CuO/Fe_3_O_4_. Cu–O of CuO was detected by deconvolution of O1s signals, with a binding energy of 530.7 eV. CuO/Fe_3_O_4_ NCs surface chemisorbed oxygen and the hydroxyl group of biomolecular capping may be the cause of the peak at 532.1 eV [68].

### 3.7 MTT-assay

**Fig. 7:**
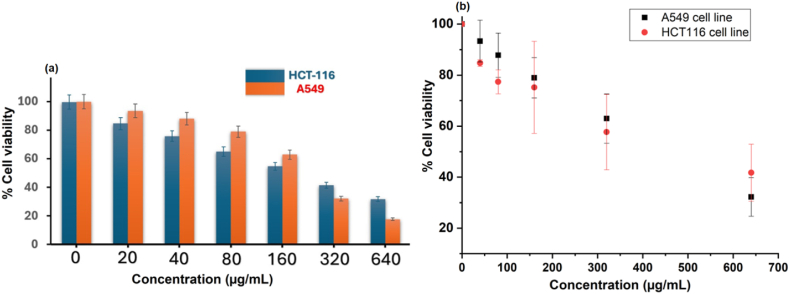
(a) The MTT-assay of the nanocomposite CuO/Fe_3_O_4_ NCs was performed against HCT-116 cell lines and A549 cell lines at the concentration range 0–640 μg/ML (b) Linear regression was performed satisfactorily and significance levels were assessed by one-way analysis of variance (ANOVA). Results are expressed as mean ± standard deviation (*n* = 3). Significance is given by one star (*) for p-values less than 0.05; two stars (**) indicate a p-value less than 0.01; and three stars (***) indicate a p-value less than 0.001. No star is used for p-values greater than or equal to 0.05, indicating non-significance.

The toxicity of the nanocomposite material was determined against cancerous cell A549 (human lung adenocarcinoma cell) and lines HCT-116(Colon cancer cell) and by MTT-Assay. It is a quite important dye and is broken down by the succinate dehydrogenase enzyme of mitochondrial living cells to produce water-insoluble purple formazan crystals. The MTT-assay of the nanocomposite material CuO/Fe_3_O_4_ NCs was carried out at 0–640 μg/ML. The IC_50_ value of the material against HCT-116 cancerous cell lines was found to be (IC_50_ = 199 μg/mL). However, the same materials displayed lower activity against A549 cancerous cell lines (IC_50_ = 445 μg/mL). Hence, the nanocomposite material exhibited better activity against HCT-116 cell lines than A549 cancerous cell lines. The MTT-assay of the nanocomposite material has been shown in Fig. 7(a and b). In Fig. 7(b), the determination of IC_50_ values involved the application of linear regression analysis as reported by similar studies [69,70]. To establish statistical significance, defined as p < 0.05, a one-way analysis of variance (ANOVA) was employed. The presentation of results incorporates the mean ± standard deviation from three independent studies (n = 3).

## 4 Conclusions

We conclude that the CuO/Fe_3_O_4_ NCs nanocomposite material was developed by biogenic synthesis (green route). The structure elucidation of material was carried by different techniques as mentioned above. The toxicity profile was evaluated against cancerous cell lines HCT-116 and A549 cells and IC_50_ values were found to be 199 μg/mL and 445 μg/mL. Thus, the nanocomposite material showed better efficacy against HCT-116 than A549 cell lines.

## Funding

The authors extend their appreciation to 10.13039/501100002383King Saud University for funding this work through research supporting project (RSP2024R376), Riyadh, Saudi Arabia.

## Data availability statement

Data will be made available on request.

## CRediT authorship contribution statement

**Poonam Dwivedi:** Methodology, Formal analysis, Data curation, Conceptualization. **Abdul Malik:** Supervision, Funding acquisition, Conceptualization. **Hafiza Zumra Fatima Hussain:** Writing – review & editing. **Indu Jatrana:** Methodology, Investigation, Conceptualization. **Khalid Imtiyaz:** Validation, Software, Formal analysis. **M.M. Alam Rizvi:** Software, Resources, Investigation. **Md Mushtaque:** Writing – original draft, Data curation. **Azhar U. Khan:** Writing – original draft, Project administration, Conceptualization. **Mahboob Alam:** Writing – review & editing. **Mohd Rafatullah:** Writing – review & editing, Supervision, Conceptualization.

## Declaration of competing interest

The authors declare that they have no known competing financial interests or personal relationships that could have appeared to influence the work reported in this paper.
